# Colorectal cancer-associated mutations impair EphB1 kinase function

**DOI:** 10.1016/j.jbc.2023.105115

**Published:** 2023-07-30

**Authors:** Yunyoung Kim, Sultan Ahmed, W. Todd Miller

**Affiliations:** 1 Department of Physiology and Biophysics, Stony Brook University, Stony Brook, New York, USA; 2 Department of Veterans Affairs Medical Center, Northport, New York, USA

**Keywords:** receptor tyrosine kinase, enzyme mutation, cell compartmentalization, cell migration, cell signaling, colorectal cancer, enzyme kinetics, protein purification, protein stability

## Abstract

Erythropoietin-producing hepatoma (Eph) receptor tyrosine kinases regulate the migration and adhesion of cells that are required for many developmental processes and adult tissue homeostasis. In the intestinal epithelium, Eph signaling controls the positioning of cell types along the crypt-villus axis. Eph activity can suppress the progression of colorectal cancer (CRC). The most frequently mutated Eph receptor in metastatic CRC is EphB1. However, the functional effects of EphB1 mutations are mostly unknown. We expressed and purified the kinase domains of WT and five cancer-associated mutant EphB1 and developed assays to assess the functional effects of the mutations. Using purified proteins, we determined that CRC-associated mutations reduce the activity and stability of the folded structure of EphB1. By mammalian cell expression, we determined that CRC-associated mutant EphB1 receptors inhibit signal transducer and activator of transcription 3 and extracellular signal-regulated kinases 1 and 2 signaling. In contrast to the WT, the mutant EphB1 receptors are unable to suppress the migration of human CRC cells. The CRC-associated mutations also impair cell compartmentalization in an assay in which EphB1-expressing cells are cocultured with ligand (ephrin B1)-expressing cells. These results suggest that somatic mutations impair the kinase-dependent tumor suppressor function of EphB1 in CRC.

Erythropoietin-producing hepatoma (Eph) receptors constitute the largest known family of receptor tyrosine kinases in humans with 14 receptors classified into type A and type B (1, 2). Activation of Eph receptors is dependent on the binding of their cognate membrane-tethered ephrin (Efn) ligands. Human EphA receptors (EphA1–A8 and EphA10) preferentially bind to glycophosphatidylinositol-linked EfnA ligands, whereas human EphB receptors (EphB1–B4 and EphB6) bind to transmembrane EfnB ligands (3, 4, 5).

EphB receptors and EfnB ligands are important in the homeostasis of the gastrointestinal tract; signaling regulates cell sorting in the mature epithelium (6, 7, 8). There is a distinct expression gradient of EphB receptors and EfnB ligands in the colon crypt axis (9). EphB receptors are expressed at the base of the crypt, while EfnB ligands are expressed at the top of the crypt. EphB activation leads to repulsion responses, resulting in segregation and proper positioning of the cells expressing EphB receptors and cells expressing EfnB ligands (10). Furthermore, repulsive interactions between EphB-positive tumor cells and EfnB-positive normal cells restrict the expansion of incipient adenomas and suppress tumor progression (11).

Eph receptors can have a dual role in tumorigenesis leading to tumor promotion or tumor suppression depending on the cellular context, the specific receptor involved, and other factors that are not well understood (12, 13). Genetic screens of cancer specimens and cell lines have identified somatic mutations in nearly all Eph receptors across various cancer types (13). EphB receptors play a tumor suppressor role in colorectal cancer (CRC), and the extent of EphB downregulation correlates with poor prognosis (14, 15). Reduced expression of EphB1 in CRC is associated with poor differentiation and increased invasive potential (16). In addition, EphB1 is frequently mutated in CRC, with particularly high incidence in metastatic CRC, suggesting that EphB1 mutations might have clinical relevance in predicting the development of metastatic disease (17). However, the biological significance of a majority of EphB1 mutations has not been studied. In one study, point mutations in the extracellular and kinase domains of EphB1 diminished the sorting and compartmentalization capacity of CRC cells (17).

To gain insight into the mechanism by which somatic mutations impair EphB1-mediated tumor suppression, we assessed their effects on kinase structure and function, using *in vitro* assays with purified proteins and by expression in mammalian cells. We discovered that four CRC-associated mutations alter EphB1 activity, stability, cell signaling, cell migration, and cell compartmentalization. These results help to explain the disruption of tumor suppressor function observed in CRC with EphB1 mutations.

## Results

### CRC-associated EphB1 mutations decrease kinase activity

Relatively little biochemical work has previously been carried out on purified EphB1. We established a protein expression and purification scheme for the kinase domain of WT EphB1. We coexpressed the His-tagged EphB1 kinase domain with YopH phosphatase and GroEL chaperone in *Escherichia coli* BL21DE3 cells (18, 19) and purified the protein by nickel-nitrilotriacetic acid chromatography (Fig. S1). The purified WT EphB1 kinase domain is highly active, and the protein retains the enzymatic activity after extended storage at −80 °C. We expressed and purified five cancer-associated mutants using a similar strategy. All of the mutants were expressed in bacteria at similar levels as the WT with the exception of the R743W mutant, which was expressed at a lower level. Nonetheless, we were able to produce sufficient quantities of all five mutants for further characterization (Fig. S1).

**Figure 1:**
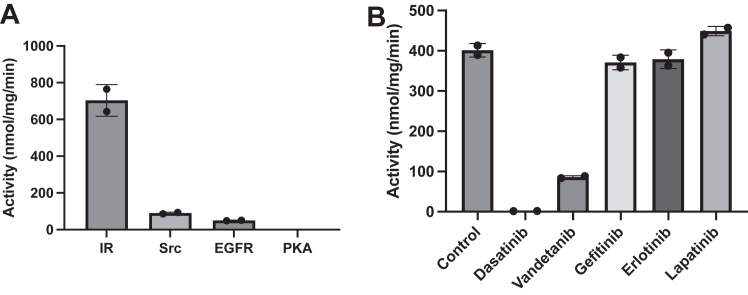
**Activity of WT EphB1**. *A*, the activities of WT EphB1 (100 nM) toward synthetic peptides containing recognition motifs for several kinases at 10 min were measured using the phosphocellulose binding assay. The error bars represent standard deviations. *B*, the activities of WT EphB1 (100 nM) against FDA-approved small molecule tyrosine kinase inhibitors at 10 min were measured using the phosphocellulose binding assay with the IR peptide. The proteins were preincubated with 1% DMSO (control) or 1 μM inhibitor for 10 min. The error bars represent standard deviations. DMSO, dimethylsulfoxide; Eph, erythropoietin-producing hepatoma; FDA, Food and Drug Administration; IR, insulin receptor.

Using a phosphocellulose binding assay (20) with [γ-^32^P]-ATP, we screened synthetic peptides containing recognition motifs for several kinases as potential substrates of the WT EphB1 kinase. We identified the insulin receptor (IR) peptide as a good *in vitro* substrate for measuring EphB1 activity (Fig. 1*A*). Next, we screened Food and Drug Administration-approved small molecule tyrosine kinase inhibitors against the WT EphB1 kinase. The multikinase inhibitor dasatinib completely blocked EphB1 activity (Fig. 1*B*). The vascular endothelial growth factor receptor 2 inhibitor vandetanib also caused a significant reduction in activity, while the epidermal growth factor receptor inhibitors gefitinib, erlotinib, and lapatinib were ineffective.

**Figure 2:**
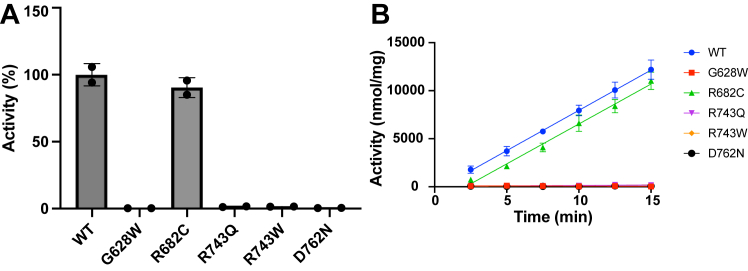
**Activities of WT and cancer-associated mutant EphB1.***A*, the activities of WT and cancer-associated mutant EphB1 (100 nM) were measured using the phosphocellulose binding assay with the IR peptide. The relative activities after 15 min of reaction were plotted as a bar graph. The error bars represent standard deviations. *B*, the activities of WT and cancer-associated mutant EphB1 (100 nM) at the indicated time points were measured using the phosphocellulose binding assay. The error bars represent standard deviations. Eph, erythropoietin-producing hepatoma; IR, insulin receptor.

The G628W, R682C, R743Q, R743W, and D762N mutations are found in the kinase domain of EphB1. The functional effects of the G628W mutation on EphB1 signaling have previously been studied in CRC cells (17). The other four (R682C, R743Q, R743W, and D762N) are the most frequently occurring EphB1 kinase domain mutations identified in the Catalogue of Somatic Mutations in Cancer database. Using the phosphocellulose binding assay with the IR peptide, we measured the activities of the WT and five cancer-associated mutant EphB1 kinases (Fig. 2). The G628W, R743Q, R743W, and D762N mutants had an almost complete loss of activity, whereas the R682C mutant had a similar level of activity as the WT. The four loss-of-function mutations (G628W, R743Q, R743W, and D762N) are all associated with CRC. In contrast, the R682C mutation has not been observed in CRC. R682C is associated with endometrioid carcinoma, adult T cell lymphoma-leukemia, and malignant melanoma, but the role of EphB1 in these cancer types has not been well studied.

### CRC-associated EphB1 mutations decrease protein stability

**Figure 3:**
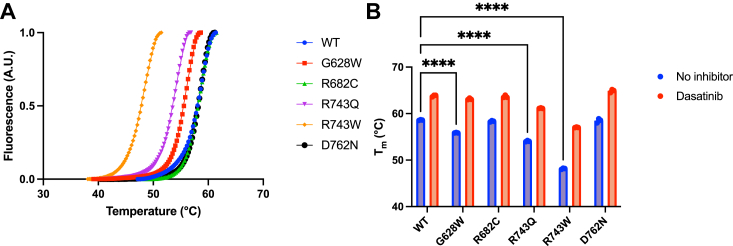
**Thermal melt analysis of WT and cancer-associated mutant EphB1.***A*, the triplicate melt curves of WT and cancer-associated mutant EphB1 were averaged and fit to the Boltzmann sigmoidal equation. *B*, the T_m_ of WT and cancer-associated mutant EphB1 in the absence and presence of dasatinib were derived from the Boltzmann sigmoidal equation. The error bars represent standard deviations (∗∗∗∗: <0.0001). Eph, erythropoietin-producing hepatoma.

Point mutations often result in a reduction of protein stability (21). To assess whether the cancer-associated mutations affected the stability of the folded structure of EphB1, we carried out a thermal shift assay. The T_m_ is the temperature at which 50% of the protein is denatured (22). The G628W, R743Q, and R743W mutants had lower T_m_ than the WT, suggesting changes in the thermodynamic stability of EphB1 (Fig. 3*A*). The lower T_m_ for the R743W mutant is consistent with the lower level of expression in bacteria noted above. The R682C and D762N mutants had similar T_m_ as the WT.

Most ligands stabilize proteins upon binding causing an increase in T_m_ (23). To assess whether the mutations affected ATP binding, we repeated the experiments in the presence of ATP-competitive inhibitor dasatinib, which bound well to the WT EphB1 kinase (Fig. 1*B*). The increased T_m_ in all five cancer-associated mutants indicated the presence of well-formed ATP binding sites in the EphB1 kinases (Figs. 3*B* and S2).

### CRC-associated EphB1 mutations decrease catalytic efficiency

**Figure 4:**
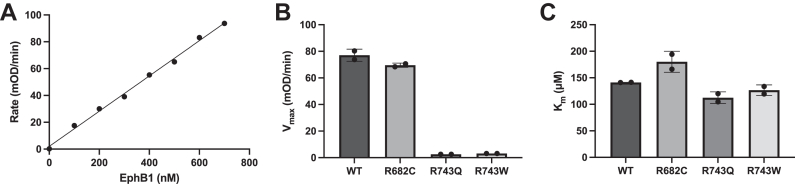
**Steady-state kinetic analysis of WT and cancer-associated mutant EphB1**. *A*, the initial rates of WT EphB1 at the indicated enzyme concentrations with 1 mM ATP were measured using the continuous spectrophotometric assay and fit to a linear regression. *B*, the *V*_max_ values of WT and cancer-associated mutant EphB1 (500 nM) were derived from the Michaelis–Menten equation. The error bars represent standard deviations. *C*, the *K*_m_ (ATP) values of WT and cancer-associated mutant EphB1 (500 nM) were derived from the Michaelis–Menten equation. The error bars represent standard deviations. Eph, erythropoietin-producing hepatoma.

To measure the steady-state kinetic values of the WT and cancer-associated mutant EphB1 kinases, we carried out a continuous spectrophotometric assay (24). In this assay, the production of ADP is enzymatically coupled to the oxidation of NADH, which is measured as a reduction in absorbance at 340 nm. The assay showed the expected linear relationship between activity and concentration of the WT EphB1 kinase (Fig. 4*A*). The G628W and D762N mutants did not have any detectable activity in this assay. The R743Q and R743W mutants had significantly reduced *V*_max_ values compared to the WT (Figs. 4*B* and S3). The R682C mutant had a similar *V*_max_ value as the WT, consistent with the results of the phosphocellulose binding assay. The R682C, R743Q, and R743W mutants had similar *K*_m_ values for ATP as the WT (Figs. 4*C* and S3). These results suggest that the R743Q and R743W mutations primarily affect the turnover rate and catalytic efficiency of EphB1.

### CRC-associated EphB1 mutations decrease Stat3 and Erk1/2 signaling

**Figure 5:**
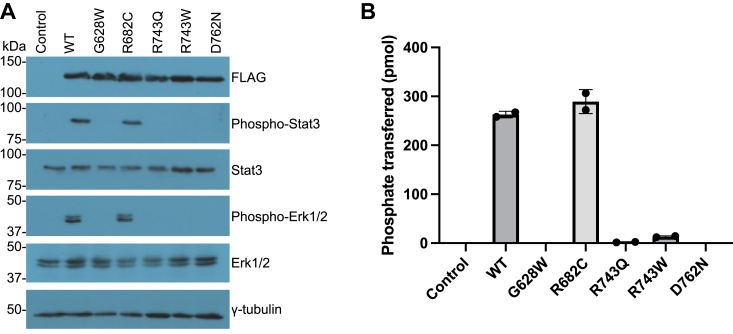
**Signaling and activities of WT and cancer-associated mutant EphB1 in HEK293T cells**. *A*, the phosphorylation of Stat3 and Erk1/2 in FLAG-tagged WT and cancer-associated mutant EphB1-expressing HEK293T cells were analyzed by Western blotting. The control is nontransfected cells. The figure is representative of two independent experiments. *B*, the activities of WT and cancer-associated mutant EphB1 immunoprecipitated from HEK293T cells were measured using the phosphocellulose binding assay with the IR peptide. The error bar represents standard deviations. Eph, erythropoietin-producing hepatoma; Erk1/2, extracellular signal-regulated kinases 1 and 2; HEK293T, human embryonic kidney 293T; IR, insulin receptor; Stat3, signal transducer and activator of transcription 3.

The enzymatic assays described above were carried out on the isolated kinase domains of EphB1. To assess the effects of the cancer-associated EphB1 mutations in mammalian cells, we expressed the full-length WT and five cancer-associated mutant EphB1 receptors in human embryonic kidney 293T (HEK293T) cells. All forms of EphB1 were expressed well in these cells (Fig. 5*A*), including the R743W mutant, which was expressed at a lower level in bacteria and had reduced thermal stability (Fig. 3). Thus, regions outside the kinase domain may be required to yield high expression. Alternatively, high expression of the proteins in mammalian cells may mask small differences in expression between the WT and mutants. We immunoprecipitated the FLAG-tagged EphB1 receptors and measured their activities using the phosphocellulose binding assay with the IR peptide (Fig. 5*B*). Consistent with the enzymatic data, the R682C mutant had a similar level of activity as the WT. The G628W and D762N mutants had a complete loss of activity, similar to the nontransfected control, while the R743Q and R743W mutants had small amounts of residual activity.

Efn-induced dimerized Eph receptors can oligomerize to form clusters, from which signaling proteins can be recruited, phosphorylated, and activated (2). However, when Eph receptors are highly expressed, they can exhibit basal activity even in the absence of Efn stimulation (2, 25, 26). EphB1 is known to activate the signal transducer and activator of transcription 3 (Stat3) and extracellular signal-regulated kinase 1 and 2 (Erk1/2) pathways (27, 28). Therefore, we probed for basal-level Stat3 and Erk1/2 phosphorylation in HEK293T cells expressing the WT and mutant EphB1 receptors using phospho-specific antibodies (Fig. 5*A*). The cells expressing the CRC-associated mutants had reduced levels of Stat3 and Erk1/2 phosphorylation, while the R682C mutant had a similar level as the WT. A similar pattern of signaling was observed when the cells were stimulated with preclustered EfnB2-Fc (Fig. S4), suggesting that the concentration of EphB1 in the plasma membrane was sufficient to form clusters without Efn ligands.

### CRC-associated EphB1 mutations do not suppress cell migration

**Figure 6:**
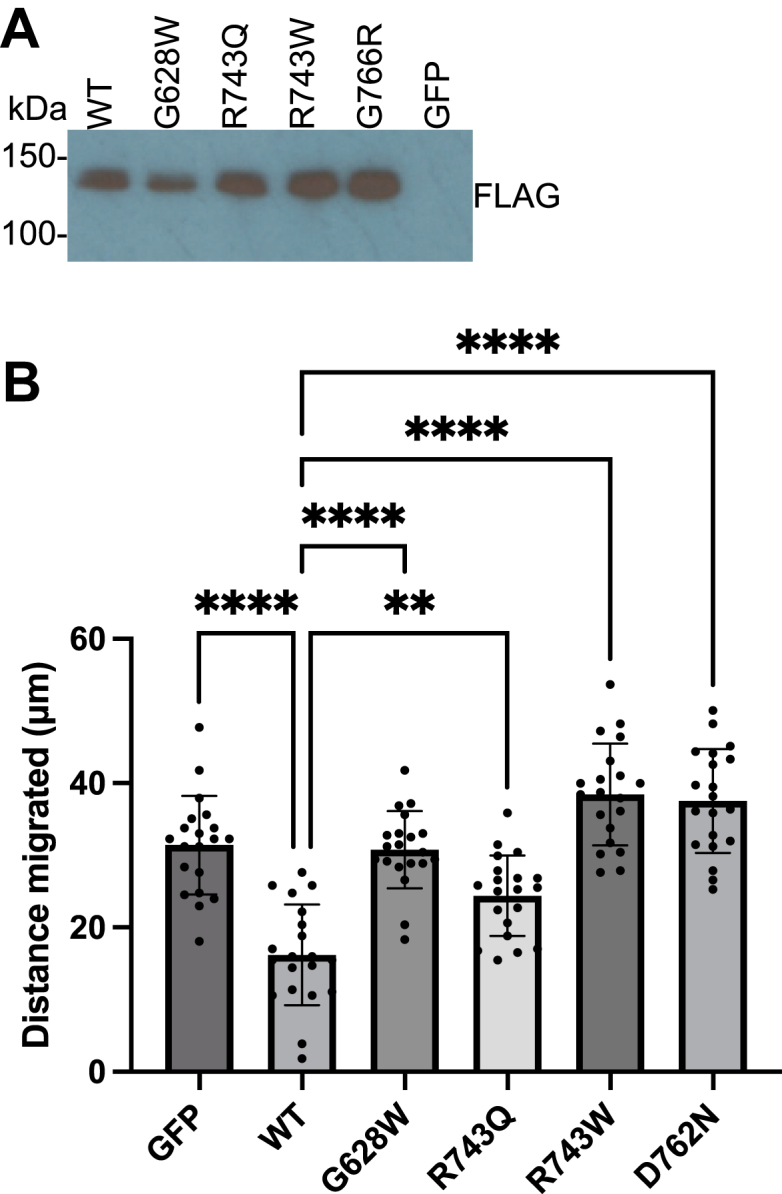
**Migration of DLD1 cells expressing WT and CRC-associated mutant EphB1**. *A*, the expression of FLAG-tagged WT and CRC-associated mutant EphB1 in DLD1 cells were probed with FLAG antibody. *B*, five random distances across the wounds were measured on day 0 and day 2. The differences between the measurements from day 0 and day 2 were plotted. The error bars represent standard deviations (∗∗∗∗: <0.0001; ∗∗: <0.01). CRC, colorectal cancer; Eph, erythropoietin-producing hepatoma.

Cell migration and cell fate within the intestine are regulated by counter gradients of EphB receptors and EfnB ligands, which exert their effects through contact-mediated cell repulsion (10). To assess the effects of the CRC-associated mutations in this more physiologically relevant cellular context, we used retroviral expression to generate stable DLD1 human CRC cells coexpressing full-length EphB1 and GFP (Fig. 6*A*). We determined that the growth rates of cells expressing the WT and CRC-associated mutant EphB1 receptors were similar for up to 72 h (Fig. S5). We conducted a wound-healing migration assay to compare the migratory phenotype of the WT and CRC-associated mutant EphB1 receptors (Figs. 6*B* and S6). The cells expressing the WT showed a significant reduction in wound closure after 2 days compared to the GFP control. However, the cells expressing CRC-associated mutants showed increased migration compared to the WT, similar to the GFP control, suggesting that EphB1 activity plays a role in regulating the migration of DLD1 cells.

### CRC-associated EphB1 mutations impair cell compartmentalization

The compact alignment of the colon crypts forces continuous repulsive interactions between EphB-positive tumor cells and EfnB-positive normal cells, which block the ability of EphB-positive tumor cells to invade EfnB-positive territories (11). *In vitro*, EfnB1 activation of EphB1 induced sorting and compartmentalization of DLD1 cells, while a mutation in the extracellular fibronectin type III domain and a mutation in the kinase domain (G628W) diminished this phenotype (17). As described above, we have confirmed that the phenotype of the G628W mutant is due to the loss of EphB1 activity (Figs. 2 and 5*B*).

**Figure 7:**
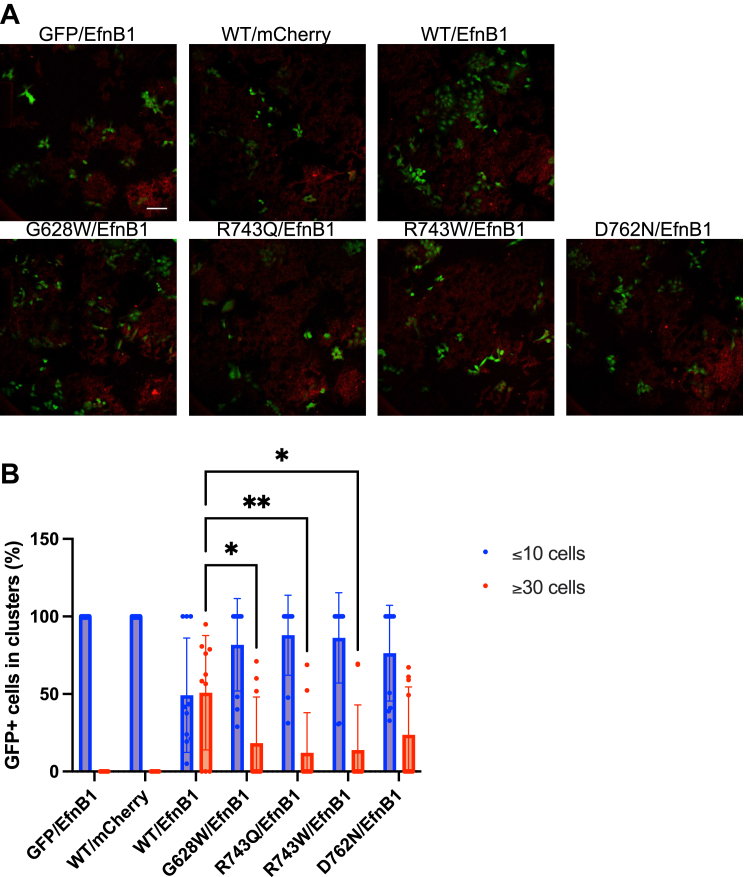
**Compartmentalization of DLD1 cells expressing WT and CRC-associated mutant EphB1.***A*, the representative fluorescence images of DLD1 cells expressing WT and CRC-associated mutant GFP-EphB1 cocultured with DLD1 cells expressing mCherry-EfnB1 were taken at 20× magnification (the scale bar represents 100 μm). *B*, the cell distribution was quantified by counting the percentage of GFP+ cells forming clusters of different sizes. GFP/EfnB1 indicates GFP-expressing DLD1 cells cocultured with mCherry-EfnB1-expressing DLD1 cells. WT/mCherry indicates GFP-WT EphB1-expressing DLD1 cells cocultured with mCherry-expressing DLD1 cells. The error bars represent standard deviations (∗∗: <0.01; ∗: <0.05). CRC, colorectal cancer; Eph, erythropoietin-producing hepatoma; Efn, ephrin.

To assess the effects of additional loss-of-function mutations studied here (R743Q, R743W, and D762N) on EphB1-mediated DLD1 cell compartmentalization, we carried out coculture compartmentalization assays (Fig. 7). In the absence of EphB1-EfnB1 interactions (GFP/EfnB1 and WT/mCherry in Fig. 7), the majority of GFP+ cells form small clusters of fewer than 10 cells. In contrast, in the WT/EfnB1 coculture, a higher percentage of GFP+ cells were distributed into large homogenous clusters (≥30 cells). Consistent with the results of the previous study, in the G628W/EfnB1 coculture, there was a reduction in the number of GFP+ cells forming large homogenous clusters compared to the cell distribution in the WT/EfnB1 coculture. There were also significant differences in the frequency of cells distributed in large clusters between the WT/EfnB1 and G628W/EfnB1, R743Q/EfnB1, and R743W/EfnB1 cocultures. The results of this assay, performed using EfnB1-expressing cells, are consistent with the results above including the enzymatic assays of the kinase domain and other cell assays without any ligand stimulation. Together, these results demonstrate that the modulation of EphB1 function by somatic mutations can impair its tumor suppressor properties.

## Discussion

The kinase domain is a key target of cancer mutations (29, 30). By identifying groups of coevolving residues, the kinase domain can be organized into three sectors that encode distinct functions: catalysis, substrate specificity, and regulation (31). The catalytic sector is most highly conserved, and it encompasses known kinase architectural determinants that are important for catalytic transfer of phosphate from ATP to a substrate hydroxyl group. Some of these determinants include glycine residues in the glycine-rich loop, the catalytic loop HRD motif, and the activation loop DFG motif (31, 32, 33). The catalytic sector is also significantly enriched for somatic cancer mutations (31).

The CRC-associated mutations studied here fall in key catalytic sector residues. Residue G628 is located in the glycine-rich loop, R743 is in the HRD motif, and D762 is in the DFG motif, and they are all highly conserved among human kinases (Fig. S7). The glycine-rich loop contributes to coordination of the phosphates of ATP (32, 34, 35, 36). This is also the most flexible part of the N-terminal lobe; it folds over the nucleotide, positioning the γ-phosphate of ATP for catalysis. Even relatively modest mutations in the glycine-rich loops of kinases can affect ATP and/or peptide binding and cause significant reductions in the rates of phosphoryl transfer (37). Therefore, the insertion of a bulky Trp residue into the glycine-rich loop for the G628W mutant would be expected to impair substrate binding. The arginine in the HRD motif coordinates with the phosphorylated tyrosine in the activation loop and allows allosteric coupling between the regulatory site and the active site (32, 38, 39, 40). Mutating R743 to an uncharged glutamine or tryptophan may have disrupted the electrostatic interactions that sustain kinase activity and stability. The aspartate in the DFG motif is important for catalysis: it binds the Mg^2+^ ions that coordinate the β- and γ-phosphates of ATP (36, 41). Mutation of the DFG Asp residue abolishes activity in many kinases (42). The loss of kinase activity we observed for the D762N mutant is consistent with a crucial role for the DFG Asp residue in EphB1 nucleotide binding and catalysis.

The G628W, R743Q, R743W, and D762N mutant EphB1 kinases all bound dasatinib, suggesting that ATP binding was intact (Figs. 3*B* and S2). On the other hand, the *V*_max_ values were significantly reduced for the R743Q and R743W mutants and undetectable for the other two CRC-associated mutant EphB1 kinases (Fig. 4*B*). Therefore, their loss-of-activity phenotypes are due to the disruption of critical components in the catalytic core of the EphB1 kinase domain. The expression and assay approaches we described here should be applicable to studies of other cancer-associated EphB1 mutations, although a limitation is that they would be restricted to mutations occurring within the kinase domain.

A limitation of our work is that these experiments do not resolve the roles of these inactivating mutations in patients. Mutations in several Eph receptors suppress the malignant properties of CRC and other cancer cells (13, 17). However, studies of mutant Eph receptors have shown that they lack the properties of classical tumor suppressors (*i.e.*, they do not require inactivation of both alleles to affect tumor progression). This could be explained by dominant negative or heterodimerization effects (43). For example, EphA and EphB receptors can cocluster, and expression of kinase-inactive EphA3 can inhibit the function of EphB2 (44). Therefore, it is possible that the EphB1 mutants studied here could cross modulate other Eph receptors in CRC.

Similar to its role in CRC, EphB1 plays a tumor suppressor role in glioma (45). Enhanced EphB1 signaling reduces the migration and invasion of glioma, suggesting a fairly widespread role in reducing cell motility. In CRC cells, EphB signaling restricts the ability of malignant cells to expand by promoting the recruitment of E-cadherin to the membrane for cell-to-cell adhesion (11). Therefore, it is possible that the CRC-associated EphB1 mutations interfere with this process. Because signaling pathways that mediate the functional effects of Eph receptors are highly cell- and receptor-type specific, more studies are still needed to elucidate the crosstalk between EphB1 and other signaling molecules to better understand the mechanism of EphB1 regulation on tumor cell behaviors.

## Experimental procedures

### Materials

[γ-^32^P]-ATP was purchased from PerkinElmer (NEG002A100UC). The synthetic peptides were purchased from Genemed Synthesis Inc and purified by semipreparative HPLC on a Vydac C18 column. HEK293T and DLD1 cells were purchased from the American Type Culture Collection. The following antibodies were used: FLAG (Sigma-Aldrich A8592), phospho-Stat3 (Tyr705) (CST9145), Stat3 (CST4904), phospho-p44/42 MAPK (Erk1/2) (Thr202/Tyr204) (CST4370), p44/42 MAPK (Erk1/2) (CST4695), γ-tubulin (Sigma-Aldrich T6557), ECL rabbit IgG (Cytiva NA9340), and ECL mouse IgG (Cytiva NA931). The molecular weights of proteins observed with these antibodies were consistent with the values in literature. In Western blotting experiments, we performed controls by omitting the primary antibody to test for potential background caused by the secondary antibodies. For Stat3, we confirmed results by using an alternative Stat3 antibody (F-2) (sc-8019).

### Protein expression and purification

EPHB1_HUMAN_D0 was a gift from Drs John Chodera, Nicholas Levinson, and Markus Seeliger (Addgene plasmid # 79694). It contains the human EphB1 kinase domain sequence (UniProtKB: P54762–1, residues 619–882) in pET His10 TEV LIC. The mutations were introduced by QuickChange site-directed mutagenesis (Agilent) and verified by DNA sequencing. The proteins were coexpressed with YopH phosphatase and GroEL chaperone in *E. coli* BL21DE3 cells (18, 19). After IPTG induction, the cells were lysed using a French press in 20 mM Tris pH 8, 500 mM NaCl, 5% glycerol, 1 mM imidazole, 1 mM phenylmethylsulfonyl fluoride, 200 μM sodium orthovanadate, 10 μg/ml aprotinin, and 10 μg/ml leupeptin. The lysate was centrifuged at 40,000*g* for 30 min at 4 °C. The supernatant was filtered using a 5 μm syringe filter followed by a 0.8 μm syringe filter and incubated with nickel-nitrilotriacetic acid resin (Qiagen) for 1 h at 4 °C. The resin was washed with 20 mM Tris pH 8, 500 mM NaCl, and 5% glycerol on a gravity column. The proteins were eluted in increasing imidazole concentrations (50 mM, 75 mM, 100 mM, and 200 mM).

### Phosphocellulose binding assay

The activities of purified and immunoprecipitated proteins were measured by a phosphocellulose binding assay (20). The reactions contained 30 mM Tris pH 7.5, 20 mM MgCl_2_, 1 mg/ml bovine serum albumin, 80 μM sodium orthovanadate, 100 to 500 cpm/pmol [γ-^32^P]-ATP, 400 μM unlabeled ATP, and 650 μM peptide. The peptides used were the following: IR, KKEEEEYMMMMG; Src, AEEEEIYGEFEAKKKKG; epidermal growth factor receptor, AEEEEYFELVAKKKG; and protein kinase A, LRRASLG (46, 47, 48). The reactions were carried out at 30 °C and stopped by the addition of cold 10% trichloroacetic acid. The reactions were vortexed and centrifuged at 10,000*g* for 1 min. The supernatant was spotted onto 2 × 2 cm Whatman P81 paper. The papers were washed with 0.5% phosphoric acid and acetone. The incorporation of ^32^P was quantified using a scintillation counter (Hidex).

### Thermal shift assay

The protein stability and protein-ligand interactions were assessed by a thermal shift assay. In this assay, the quantum yield of the dye increases when it binds the exposed hydrophobic surfaces of the denatured proteins. Prior to the addition of 5× SYPRO Orange (Sigma-Aldrich), 2 μM EphB1 was incubated with dimethylsulfoxide or 10 μM dasatinib for 1 h. The proteins were heat denatured from 5 °C to 95 °C and fluorescence (excitation/emission 470 nm/570 nm) was recorded at 2 °C intervals in a StepOne Real-Time PCR machine (Applied Biosystems). The melt curves were normalized to obtain relative fluorescence values between 0 and 1. The normalized melt curves were fit to the Boltzmann sigmoidal equation in GraphPad Prism, version 9 (www.graphpad.com) to derive the T_m_’s (V_50_). The statistical significance was determined using a two-way ANOVA test in GraphPad Prism, version 9.

### Continuous spectrophotometric assay

The steady-state kinetic values were measured by a continuous spectrophotometric assay (24). The reactions contained 100 mM Tris pH 7.5, 10 mM MgCl_2_, 1 mM phosphoenolpyruvate, 128 units/ml pyruvate kinase, 184 units/ml lactate dehydrogenase, 200 μg/ml NADH, and different concentrations of IR peptide. The reactions were carried out at 30 °C in a final volume of 75 μl. The absorbance at 340 nm was recorded every 8 s in a plate reader (VersaMax). The initial rates were fit to a linear regression in GraphPad Prism, version 9 to determine the linear relationship between enzymatic activity and enzyme concentration. The initial rates were fit to the Michaelis–Menten equation in GraphPad Prism, version 9 to derive the *V*_max_ and *K*_m_ (ATP) values.

### DNA constructs

pFUGW EphB1 was a gift from Dr Matthew Dalva (Thomas Jefferson University) (49, 50). It contains the signal peptide sequence followed by the FLAG tag sequence, then followed by the full-length human EphB1 sequence (UniProtKB: P54762–1, residues 1–984). For HEK293T expression of EphB1, the EphB1 sequence was amplified by PCR and subcloned into pCMV-HA-AscI-SBP (51) using the AscI and EcoRI restriction sites. The mutations were introduced by QuickChange site-directed mutagenesis (Agilent) and verified by DNA sequencing. pBMN-I-GFP was a gift from Dr Garry Nolan (Addgene plasmid # 1736). For DLD1 expression of EphB1, the EphB1 sequence was amplified by PCR and subcloned into pBMN-I-GFP using the XhoI and NotI restriction sites. The mutations were introduced by QuickChange site-directed mutagenesis (Agilent) and verified by DNA sequencing. FUW-ubiquitin-EfnB1-SV40-RFP (b1R) was a gift from Dr Eduard Batlle (Addgene plasmid # 65446). For DLD1 expression of EfnB1, the EfnB1 sequence was amplified by PCR and subcloned into pBMN-I-mCherry using the XhoI and NotI restriction sites. To generate pBMN-I-mCherry, the GFP sequence in pBMN-I-GFP was replaced with the mCherry sequence. The GFP sequence in pBMN-I-GFP was excised using the NcoI and SalI restriction sites. The NcoI restriction site internal to the mCherry sequence in pmCherry-C1 (NovoPro Cat. No. V011976) was mutated by QuickChange site-directed mutagenesis (Agilent) and verified by DNA sequencing. The mCherry sequence was amplified by PCR and subcloned into pBMN-I using the NcoI and SalI restriction sites.

### Cell culture and transfections

All mammalian cells were cultured in Dulbecco’s modified Eagle’s medium at 4.5 g/L supplemented with 10% fetal bovine serum and 1× penicillin and streptomycin. HEK293T cells were transfected according to the TransIT-LT1 (Mirus) protocol with 10 or 2.5 μg of DNA. The retrovirus was generated and target cells were infected as described (52). pCI-VSVG was a gift from Dr Garry Nolan (Addgene plasmid # 1733). Phoenix Ampho cells were cotransfected with pCI-VSVG and either pBMN-I-GFP EphB1 or pBMN-I-mCherry EfnB1 using the TransIT-LT1 (Mirus) reagent and chloroquine. The retroviral supernatant was harvested 48 and 72 h after transfection and sterile filtered. The filtered retrovirus was added to DLD1 cells together with polybrene. The cells were sorted using GFP and mCherry fluorescence for EphB1- and EfnB1-expressing cells, respectively.

### Ligand stimulation

HEK293T cells were stimulated with 1 μg/ml preclustered EfnB2-Fc (R&D Systems) for 10 or 30 min. Preclustered oligomers of EfnB2-Fc were generated by preincubation of EfnB2-Fc with goat anti-human IgG (Jackson ImmunoResearch Laboratories) for 1 h 4 °C at a ratio of 1:2.

### Mammalian cell lysis

The cells were lysed in 25 mM Tris pH 7.5, 1 mM EDTA, 100 mM NaCl, 1% nonionic polyoxyethylene-40, 100 μM phenylmethylsulfonyl fluoride, 100 μM sodium orthovanadate, 5 μg/ml aprotinin, and 5 μg/ml leupeptin. The lysate was centrifuged at 15,000*g* for 10 min at 4 °C.

### Immunoprecipitation

The proteins were immunoprecipitated by incubating the lysate (1 mg) with 20 μl anti-FLAG affinity resin (Sigma-Aldrich) for 2 h at 4 °C. The resin was washed with 25 mM Tris pH 7.5, 1 mM EDTA, 100 mM NaCl, 1% nonionic polyoxyethylene-40, 100 μM phenylmethylsulfonyl fluoride, 100 μM sodium orthovanadate, 5 μg/ml aprotinin, and 5 μg/ml leupeptin.

### Growth assay

DLD1 cells expressing GFP or GFP-EphB1 were seeded at 1 × 10^4^ cells per well of a 24-well dish. Seventy-two hours after seeding, the cells were trypsinized, and the number of live cells were counted using a cell countess (Thermo Fisher Scientific).

### Wound-healing assay

DLD1 cells expressing GFP or GFP-EphB1 were seeded at 5 × 10^5^ cells per well of a 12-well dish. Twenty-four hours after seeding (day 0), each well was scratched through the center using a P200 tip. The area of interest along the wound was imaged using a brightfield microscope at 4× magnification. Seventy-two hours after seeding (day 2), the area of interest was imaged again. For each image, five random measurements were taken across the wound in units of micrometers using Adobe Photoshop. The distances were calculated by subtracting the measurements of day 2 from day 0. The statistical significance was determined using an one-way ANOVA test in GraphPad Prism, version 9.

### Coculture compartmentalization assay

The assay was performed as described (17). DLD1 cells expressing GFP or GFP-EphB1 were cocultured with DLD1 cells expressing mCherry or mCherry-EfnB1 onto a coverslip in a 6-well dish at a ratio of 1:3 and a total of 4 × 10^5^ cells. Forty-eight hours after seeding, the cells were fixed in 4% paraformaldehyde for 20 min and mounted onto slides using VectaShield mounting media. For each coverslip, five random images were taken using a fluorescence microscope at 20× magnification. Quantification was carried out as described (11, 17): the percentages of GFP+ cells in clusters of different sizes (≤10 cells and ≥30 cells) were calculated by counting the number of GFP+ cells from ten representative images from two independent experiments. The statistical significance was determined using a two-way ANOVA test in GraphPad Prism, version 9.

## Data availability

All data and supporting information are contained within this article.

## Supporting information

This article contains supporting information (53, 54).

## Conflict of interest

The authors declare that they have no conflicts of interest with the contents of this article.
