# Supplementary material for: Colorectal cancer-associated mutations impair EphB1 kinase function
Source: J Biol Chem. 2023 Jul 30;299(9):105115. doi: 10.1016/j.jbc.2023.105115 (PMC10463257; doi:10.1016/j.jbc.2023.105115)
Supplement: Supporting information [file mmc1.pdf]

Colorectal cancer-associated mutations impair EphB1 kinase function

Yunyoung Kim[1], Sultan Ahmed[1], and W. Todd Miller[1,2,*]

[1]Department of Physiology and Biophysics, Stony Brook University, Stony Brook, New York, USA

[2]Department of Veterans Affairs Medical Center, Northport, New York, USA

*For correspondence: W. Todd Miller todd.miller@stonybrook.edu

List of materials included: Figures S1-7

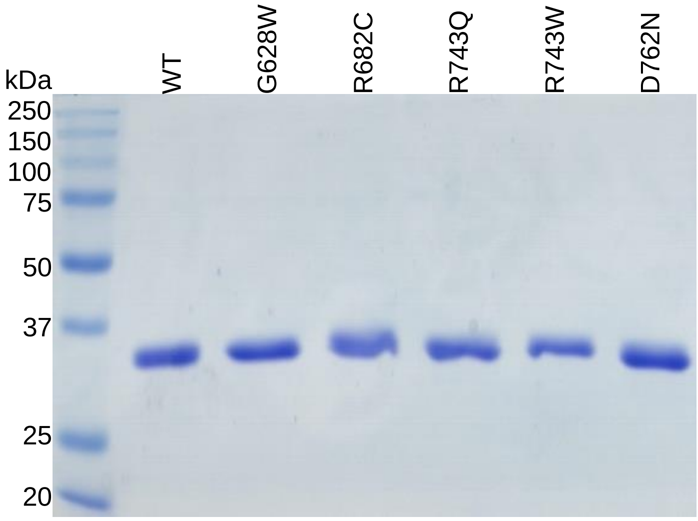

Figure S1. SDS-PAGE analysis of WT and cancer-associated mutant EphB1. The purified kinase domains (1.5 μg) were detected by Coomassie blue staining.

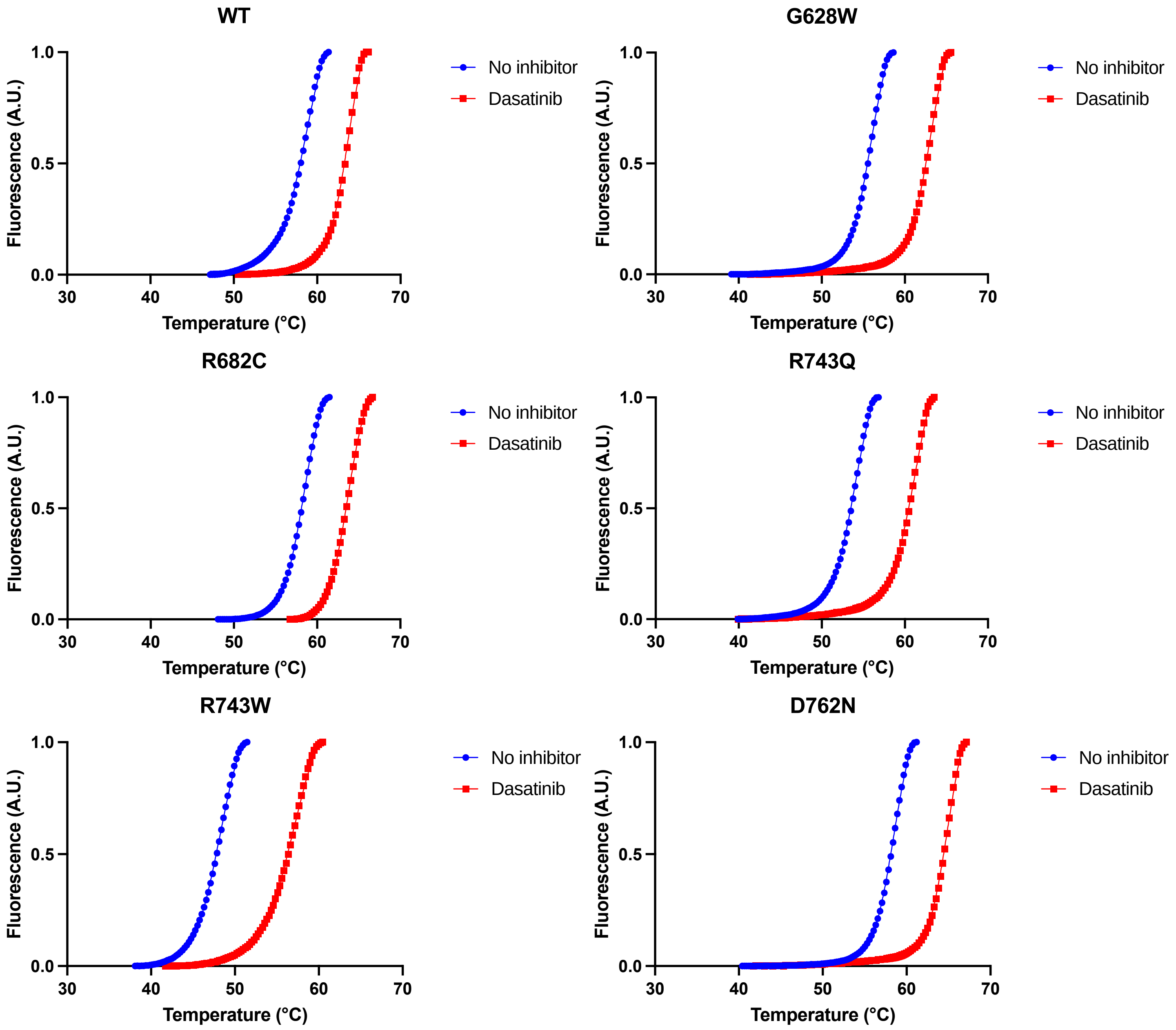

Figure S2. Thermal melt curves of WT and cancer-associated mutant EphB1. The triplicate melt curves were averaged and fit to the Boltzmann sigmoidal equation to derive the T_m's in the absence and presence of dasatinib.

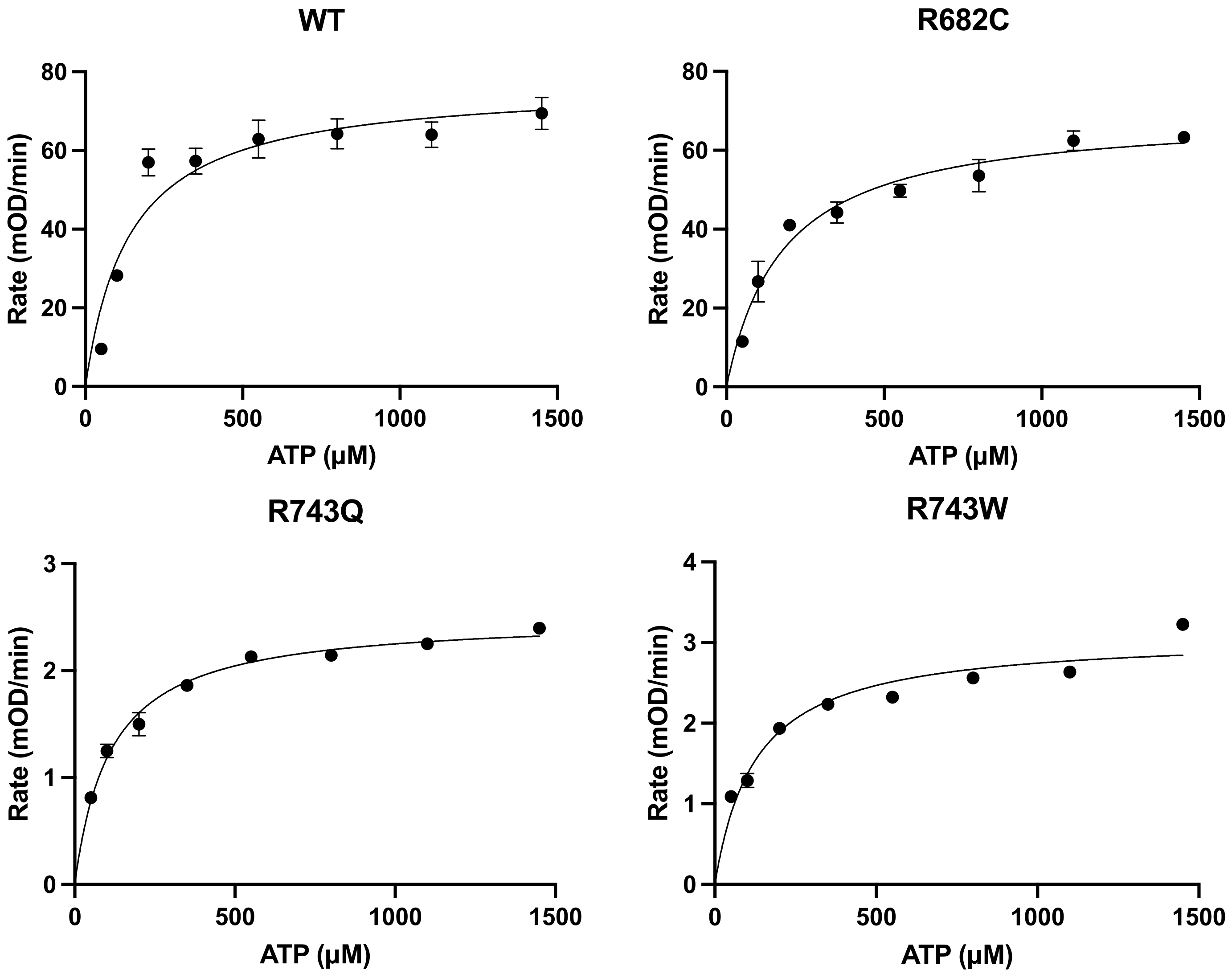

Figure S3. Michaelis-Menten plots of WT and cancer-associated mutant EphB1. The initial rates were plotted against ATP concentrations and fit to the Michaelis-Menten equation to derive the V_{max} and K_m (ATP) values. The activities were measured using the continuous spectrophotometric assay. The error bars represent standard deviations.

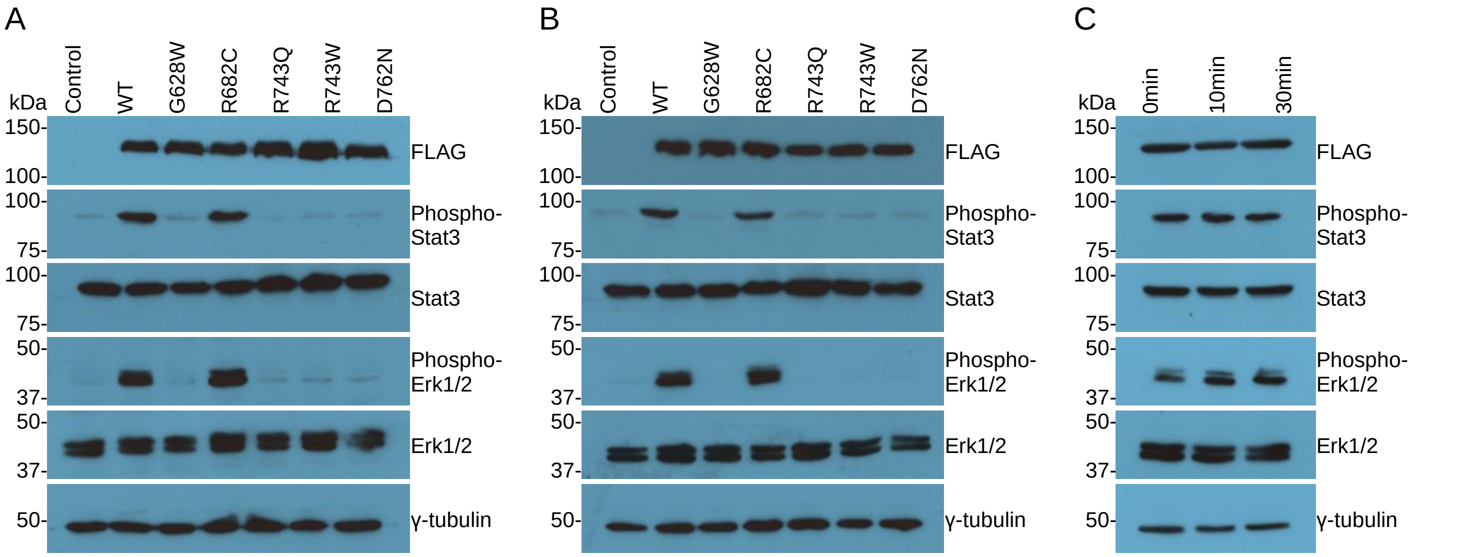

Figure S4. Ligand-stimulated signaling of WT and cancer-associated mutant EphB1 in HEK293T cells. (A) The phosphorylation of Stat3 and Erk1/2 in FLAG-tagged WT and cancer-associated mutant EphB1-expressing cells stimulated with EfnB2-Fc for 10 minutes were analyzed by Western blot. The control is non-transfected cells. (B) The phosphorylation of Stat3 and Erk1/2 in FLAG-tagged WT and cancer-associated mutant EphB1-expressing cells stimulated with EfnB2-Fc for 30 minutes were analyzed by Western blot. (C) The phosphorylation of Stat3 and Erk1/2 in FLAG-tagged WT EphB1-expressing cells stimulated with EfnB2-Fc for 0, 10, or 30 minutes were analyzed by Western blot.

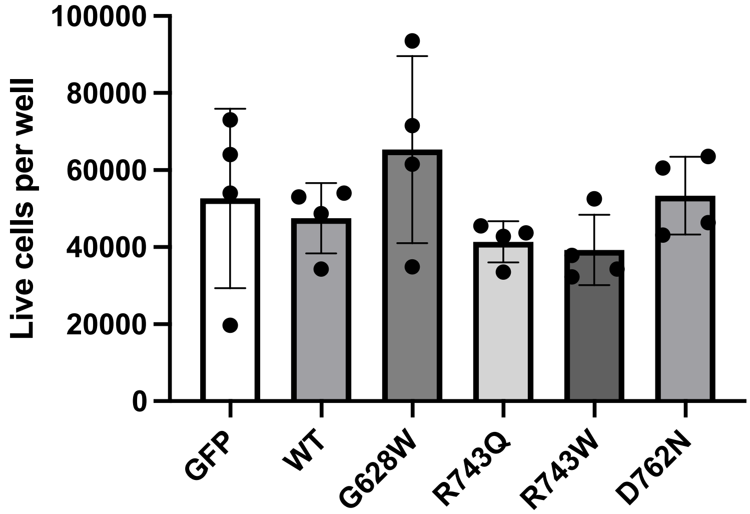

Figure S5. Growth of DLD1 cells expressing WT and CRC-associated mutant EphB1. The number of live cells were counted 72 hours after seeding. The error bars represent standard deviations.

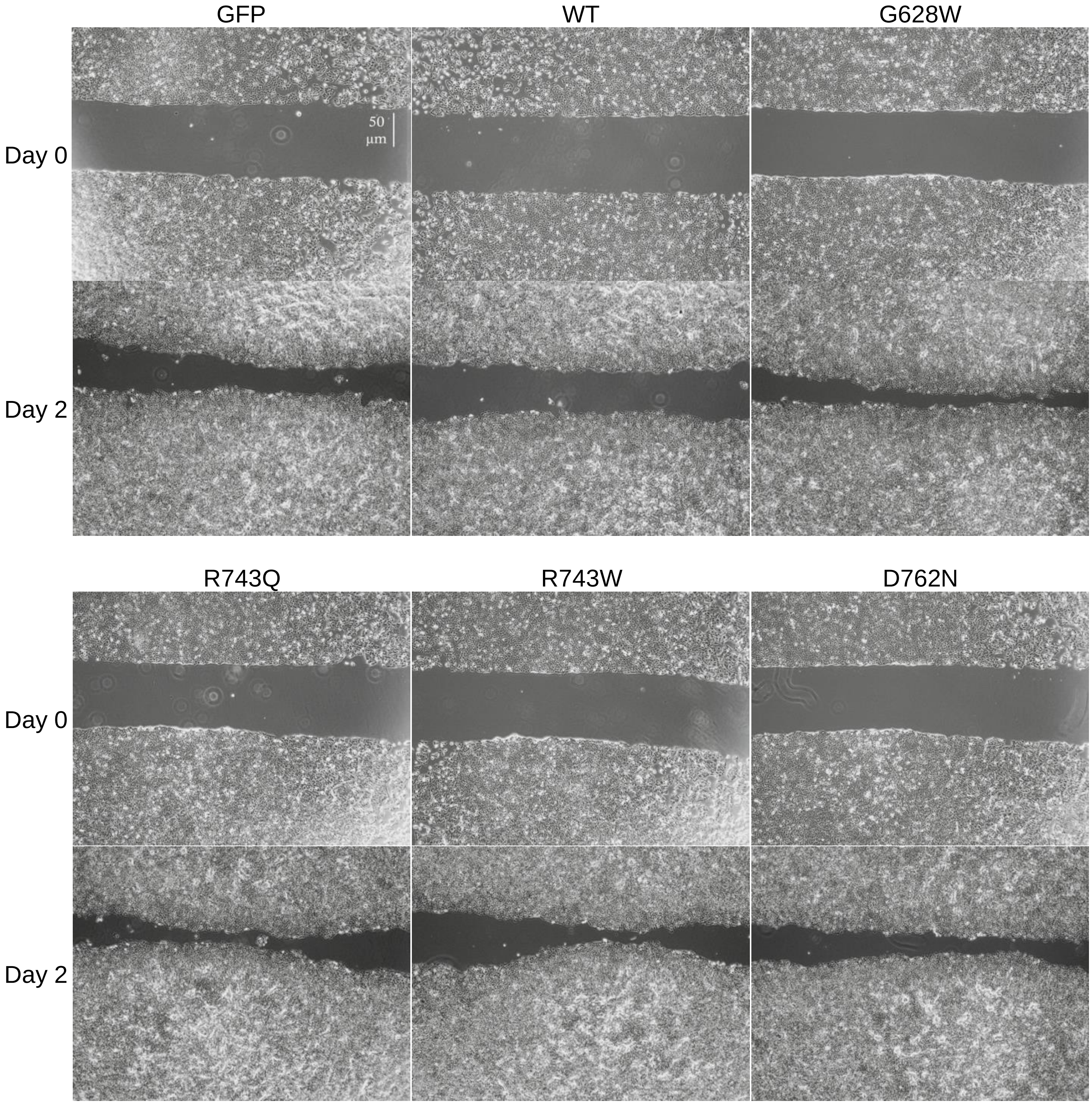

Figure S6. Representative images of wound-healing migration assay with DLD1 cells expressing WT and CRC-associated mutant EphB1. The bright-field images were taken at 4× magnification (scale bar = 50 μm).

A B

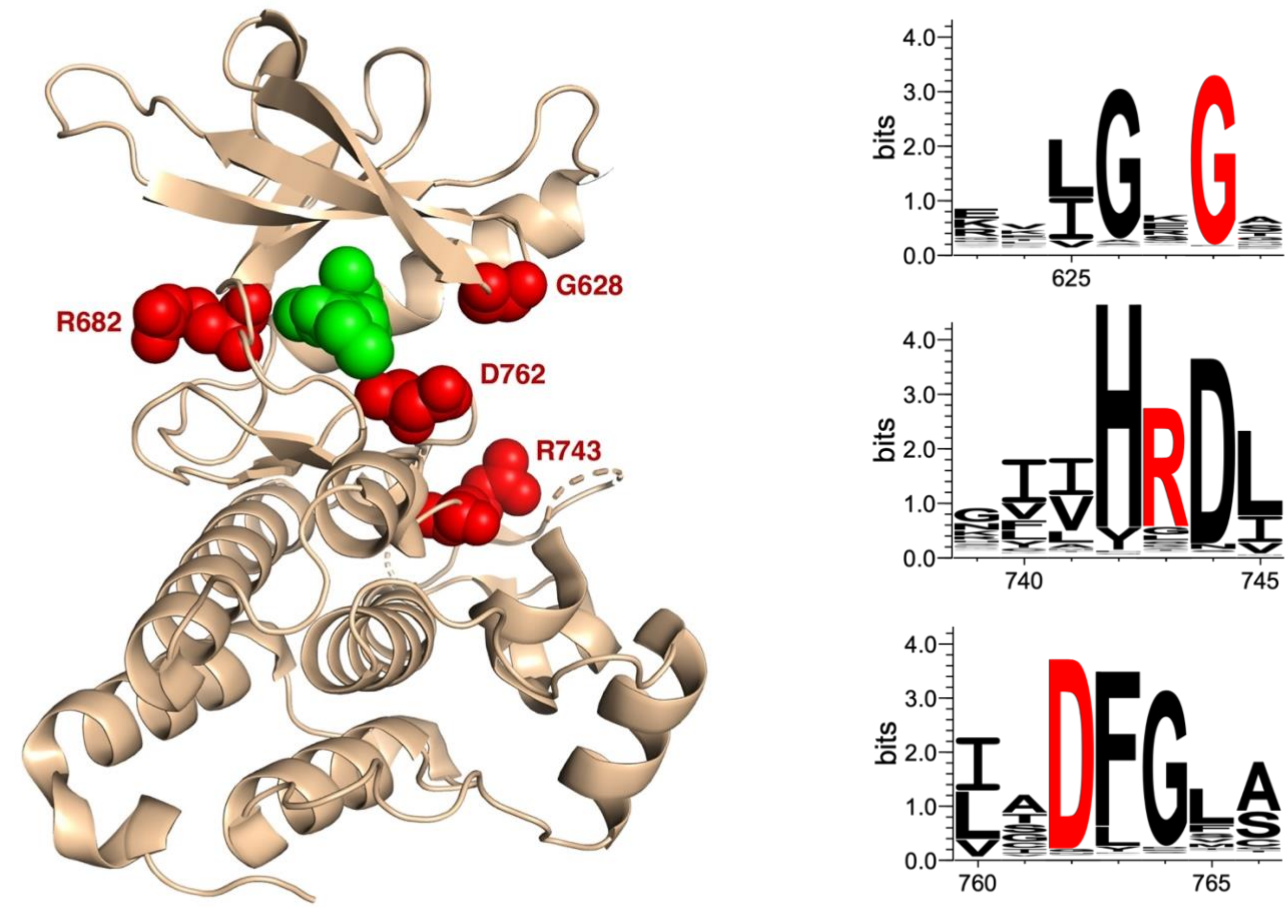

Figure S7. Cancer-associated EphB1 residues. (A) The crystal structure of human EphB1 kinase domain (PDB: 5MJA) shows the positions of cancer-associated residues in red. The quinazoline-based inhibitor is indicated in green. (B) The sequence logos show the conservation of CRC-associated EphB1 residues among 497 human kinases. The sequence logos were created with WebLogo [34] using Modi and Dunbrack's multiple sequence alignment [35] as input. The residues are numbered according to their positions in EphB1. The G628, R743, and D762 residues are indicated in red.
